# Supplementary material for: Disciplinary removal patterns among students with other health impairments and emotional disturbance: A three-year descriptive analysis of IDEA Part B data
Source: PLoS One. 2026 Apr 2;21(4):e0346338. doi: 10.1371/journal.pone.0346338 (PMC13046267; doi:10.1371/journal.pone.0346338)
Supplement: Attachment — Submitted filename: Response to Reviewer.docx [file pone.0346338.s001.docx]

Response to Reviewer #1

Manuscript ID: PONE-D-25-66721

Title: Disciplinary Removal Patterns Among Students with Other Health Impairments and Emotional Disturbance: A Three-Year Descriptive Analysis of IDEA Part B Data

We sincerely thank the reviewer for their thoughtful and constructive evaluation of our manuscript. We appreciate the positive assessment of the study’s topic and the recognition of its relevance to the education of students with disabilities. We have carefully revised the manuscript to address each concern raised. Below we respond point-by-point.

1. Clarification of Research Purpose and Precision

Reviewer Comment:

The research concept has not been described with sufficient precision. The purpose of examining patterns is not clearly articulated. Research questions are not included.

Response:

We agree that the manuscript benefited from greater precision in articulating its aims. In the revised manuscript, we have added a dedicated “Research Questions” subsection at the end of the Introduction. This section now explicitly states the guiding research questions, clarifying that the study is descriptive and comparative in nature and aimed at identifying longitudinal system-level patterns within publicly reported IDEA data.

These revisions strengthen conceptual clarity and make explicit what the analysis seeks to examine and why those patterns are relevant within policy and diagnostic contexts.

2. Description of the Dataset

Reviewer Comment:

The article lacks a detailed description of the analyzed dataset, including what the data contain, how they were created, total numbers, and procedures analyzed.

Response:

We appreciate this recommendation. The Data Source section has been substantially expanded. The revised manuscript now includes:

Identification of IDEA Section 618 Table bdiscipline-15 as the data source

Clarification that the dataset includes all 50 states and the District of Columbia

Explanation that percentages are calculated by the U.S. Department of Education using disability-specific denominators

Explicit clarification that the dataset reflects publicly reported administrative census-level data rather than a sample

Clarification that ADHD is embedded within the OHI category and not separately reported

These additions improve transparency regarding the scope, structure, and limitations of the dataset.

3. Expansion of the Analytic Approach and Statistical Rigor

Reviewer Comment:

Statistical analysis has not been performed appropriately and rigorously. The analytic process should be described in more detail.

Response:

We have expanded the Analytic Approach section to provide greater methodological transparency. Specifically, we now describe:

Inclusion of all available state-level data

Treatment of suppressed or missing values

Rationale for calculating median state-level percentages

Justification for descriptive analysis given the census-level nature of the dataset

Clarification that inferential statistical testing would not provide additional interpretive value because the dataset reflects population-level administrative reporting rather than sampled observations

The revised manuscript now more clearly explains why descriptive longitudinal analysis is methodologically appropriate for the dataset used.

4. Interpretation of ADHD-Related Findings

Reviewer Comment:

Statements relating to ADHD are too definitive given that ADHD was not analyzed separately and intervention data were not available.

Response:

We appreciate this important caution. We have revised the Discussion section to soften language where necessary and avoid overstatement. Specifically:

Definitive phrasing (e.g., “suggest a misalignment”) has been revised to conditional phrasing (e.g., “may reflect a potential misalignment”).

Statements implying intervention absence have been reframed to acknowledge that intervention data are not available in IDEA Section 618 reporting.

The Conclusion has been revised to reference students classified under OHI, rather than ADHD specifically, to maintain consistency with the available data.

These revisions ensure that interpretations remain appropriately bounded by the limitations of the dataset.

5. Placement Within Existing Literature

Reviewer Comment:

The study has not been sufficiently placed in the context of current literature.

Response:

We have expanded the Discussion to better situate the findings within existing scholarship. The revised manuscript now includes additional contextualization of prior research on:

Disciplinary exposure among students with disabilities

Differences in identification and intervention practices for OHI and ED classifications

Universal Design for Learning as a preventative instructional framework

These additions clarify how the present study contributes to the broader literature while maintaining analytic restraint.

6. Limitations and Interpretive Boundaries

We have retained and strengthened the Limitations section to emphasize:

The inability to disaggregate ADHD within OHI

The absence of intervention-level data (e.g., FBAs, BIPs)

The descriptive nature of the analysis

The inability to draw causal conclusions

We agree that conclusions must be formulated cautiously and have revised language accordingly.

Summary

We are grateful for the reviewer’s thoughtful and constructive feedback. The revisions have strengthened the manuscript by:

Clarifying research questions

Expanding methodological transparency

Refining interpretive language

Enhancing literature contextualization

We believe these changes meaningfully address the reviewer’s concerns and improve the rigor and clarity of the manuscript. We respectfully resubmit the revised version for further consideration.

Sincerely,

Melissa Beck Wells
